# Postepidemic Epidemiology of Porcine Epidemic Diarrhea Virus in the United States

**DOI:** 10.1155/2024/5531899

**Published:** 2024-10-01

**Authors:** Dennis N. Makau, Nakarin Pamornchainavakul, Kimberly VanderWaal, Mariana Kikuti, Catalina Picasso-Risso, Emily Geary, Cesar A. Corzo

**Affiliations:** ^1^ Department of Biomedical and Diagnostic Sciences College of Veterinary Medicine University of Tennessee, Knoxville, Tennessee, USA; ^2^ Department of Veterinary Population Medicine College of Veterinary Medicine University of Minnesota, Minneapolis, Minnesota, USA; ^3^ Department of Large Animal Clinical Sciences College of Veterinary Medicine Michigan State University, East Lansing, Michigan, USA

**Keywords:** endemic phase, feed mitigants, PEDV clusters, risk factor analysis

## Abstract

Porcine epidemic diarrhea virus (PEDV) emerged in the United States (U.S.) swine population in 2013, initiating an initial significant epidemic followed by a state of endemicity in the U.S. Despite continued monitoring, the epidemiology of PEDV during its endemic phase remains inadequately researched. Our study aimed to characterize the spatial–temporal distribution of postepidemic PEDV cases in the U.S. breeding herd and identify associated risk factors. Data from 1089 breeding farms in 27 states, reported to the Morrison Swine Health Monitoring Project from July 2014 to June 2021, were analyzed. We stratified the data into six U.S. regions and employed SaTScan for spatiotemporal permutation and cluster analysis. Survival analysis was used to assess risk factors. A notable seasonal clustering of PEDV cases was observed in winter (January–March; *p*=0.001, relative risk = 2.2) with regional variation. Ten high-rate spatial–temporal clusters (*p*  < 0.05) were identified ranging from 2.5 to 833.7 km^2^ and lasting 1–5 months, occurring in four regions between 2015 and 2021. For the study period, a total of 625 cases of PEDV were recorded on 372 farms. The total number of PEDV cases decreased from 95 breeding farms in 32 counties (2014–2015) to 53 farms in 28 counties (2020–2021), indicating an overall reduction in occurrence and spatial extent. Feed mitigants demonstrated a protective effect, significantly reducing the risk of PEDV occurrence (hazard ratio = 0.3, *p*=0.003), while air filtration systems exhibited marginal benefits (hazard ratio = 0.3, *p*=0.06). Other important risk factors included county farm density with farms in high-density regions (>31 farms/100 km^2^) being 1.3 times more likely to experience outbreaks than in medium-density regions (13–31 farms/1000 km^2^; *p*  < 0.001). Additionally, farms in region E had higher odds of outbreaks compared to region B. The overall decline in PEDV cases and reduced spatial extent reflect industry efforts in postepidemic control and elimination. The protective effects of feed mitigants warrant further investigation. Our findings underscore the opportunity for coordinated efforts to eliminate PEDV in the U.S. and emphasize the need for comprehensive risk profiling associated with industry practices.

## 1. Introduction

Porcine epidemic diarrhea (PED) is a swine enteric viral disease first documented in the United States (U.S.) swine population in 2013, at which time it sparked a major epidemic [1]. The clinical presentation involves high morbidity and mortality in suckling piglets with diarrhea being the main clinical sign [2, 3]. The disease is caused by a virus (PEDV) in the genus *Alphacoronavirus* [4] that is largely classified into two main genogroups, the S-INDEL strain and nonS-INDEL strain. The nonS-INDEL strain is associated with higher virulence, whereas the S-INDEL strain has multiple insertions and deletions in the spike protein region of the genome and presents with milder clinical disease. The nonS-INDEL strain was identified as the primary causative variant of the 2013–2014 PED epidemic in the U.S [5].

Since the initial epidemic, PEDV has persisted in the U.S. breeding herd with low incidence and is now considered endemic. Despite continued monitoring and surveillance, there is minimal research and epidemiological understanding of the endemic phase of PEDV in the country. At the height of the epidemic and soon after, different publications cited several risk factors associated with the rapid spread of PEDV, such as animal and farm employee movements, contamination of trucks, feed, slaughter plants, and feed-mill contamination among others [6–9]. Presently, the annual incidence in U.S. swine herds, especially the breeding population, is estimated at less than 10% with occasional increases in outbreaks that seem to fluctuate seasonally [10]. It is necessary to reevaluate mitigation measures and update stakeholders on current risks of PEDV, as well as identify changes in disease occurrence and spread that can inform considerations of the elimination of this pathogen from the U.S. swine herd.

One of the current challenges in PEDV management is the paucity of current data about the prevalence of each strain in the U.S. swine population and in which regions these strains primarily circulate in the endemic period, which contrasts with the epidemic period during which one strain dominated remains. Although personal communication from different industry veterinarians and swine producers suggests that outbreaks of PEDV continue to occur with some level of seasonality, with more cases occurring around December, there has not been recent published research investigating the postepidemic seasonality of PED outbreaks and whether these trends vary across the U.S. landscape. Additionally, the clinical picture of reported cases of PED also suggests that there could be differences in the causative strains on different farms [3, 11]. In this study we intend to address these knowledge gaps.

**Figure 1:**
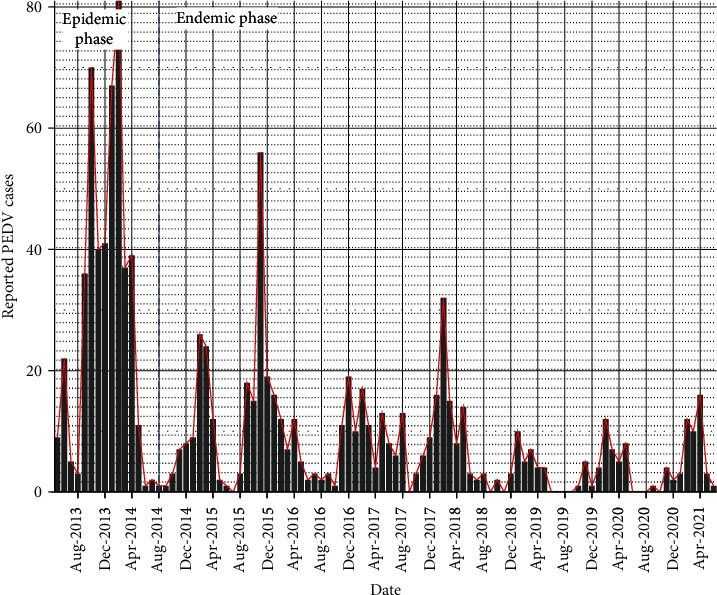
A chart depicting the number of PED cases reported in U.S. breeding farms participating in the Morison Swine Health Monitoring Project (MSHMP). The epidemic period is to the left of the blue vertical line and the post-epidemic (endemic) period is to the right of the blue line.

Biosecurity improvements in the U.S. swine industry such as feed quarantine, truck washing and disinfection, use of air filtration systems informed by studies largely performed during the epidemic (among other efforts), contributed to the successful containment of the epidemic [12]. The effect of these changes in farm biosecurity has yet to be quantified and documented for the different production phases, but one may hypothesize that risk factors related to disease introduction to farms have shifted through time due to factors found to be associated with transmission in the epidemic period were better mitigated in the endemic period. In this study, we aimed to characterize PEDV occurrence in U.S. breeding herds in the postepidemic phase highlighted in blue (Figure 1). Specifically, we investigated the spatial–temporal trends of PEDV occurrence on these herds and identified factors associated with its occurrence.

## 2. Materials and Methods

### 2.1. Study Area and Data Management

**Figure 2:**
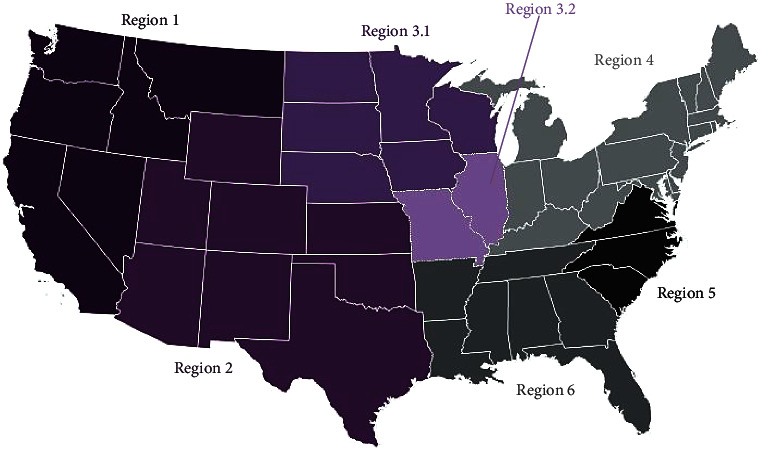
Modified stratification of study area into regions adapted from the rapid response program regional mapping of U.S. states by the Swine Health Information Center (SHIC) [13]. Five of the regions included in this analysis were randomly recoded with alphabets A–E for confidentiality purposes.

The U.S. was stratified into six regions (2, 3.1, 3.2, 4, 5, 6) based on a modified regional description adapted from the Swine Health Information Center's (SHIC) rapid response program (Figure 2) [13]. There was no data from region 1 in our dataset, reflecting low swine production in that region and one of the regions where no cases had been reported for the entire study period had disproportionately less farms (*n* = 11) and observations (4026) compared to the other regions. These two regions were excluded from the study. The remaining five regions were randomly assigned to letters A–E as per data protection and confidentiality guidelines when working with Morison Swine Monitoring Project (MSHMP) data.

In the MSHMP database, enrolled pig producing companies voluntarily report changes in PEDV infection status (i.e., outbreak, stability, and elimination) each week. Data regarding herd size, air filtration use, location, and viral pathogen sequences are also shared by participating systems upon enrollment [14, 15]. For this study, we obtained data on 1100 breeding herds that accounts for ~ 50% of the U.S. swine breeding herd. After data cleaning and restriction of study period to the postepidemic phase of PEDV (between July 2014 and June 2021; Figure 1), data from 1089 herds belonging to 36 production systems spread over 254 counties in 27 states in the U.S. were included in the study. Because both porcine reproductive and respiratory syndrome (PRRS) and PED are suggested to have seasonal increases in occurrence during winter, an epidemiological year was defined as starting in July and ending in June and thus the study period encompassed 7 epidemiological years. Of the 36 production systems, 13 provided data on the use of feed mitigants as a practice to manage PEDV on their farms. Thus, only data from these 13 systems were used in the risk factor analysis, reducing the number of observations from 86,183 farm-weeks (1089 farms) to 44,960 farm-weeks (562 farms) in 137 counties in 17 states.

From the MSHMP database, we also obtained additional farm-level attributes that were used as model covariates. These included: geographical location of the farms, whether the farms used an air filtration system (e.g., none, partial [unfiltered during summer], year-round filtration [positive or negative pressure], these were collapsed into filtered and unfiltered groups for the analysis); average inventory of the farm, which was classified based on quantiles as small (less than 2000 sows), medium (2000–3500 sows), and large (more than 3500 sows); and whether a farm used feed mitigants. Feed mitigants are organic and inorganic compounds that are added to feed prior to animal consumption for the purpose of reducing contamination by pathogens. Additionally, farm density in the county where a farm was located was calculated based on data available from USDA statistics, which was calculated as the estimated number of farms per county in 2017 [16] divided by the area of the county in square kilometers. Using quantiles, farms were then categorized as being in high, medium, or low farm-density areas, i.e., >31 farms/1000 km^2^, 13–31 farms/1000 km^2^, and <13 farms/1000 km^2^, respectively. We defined the season of outbreak occurrence conventionally as summer (June–August), fall (September–November), winter (December–February), and spring (March–May). We also obtained all PEDV sequences generated by any MSHMP participating production system at three partnering veterinary diagnostic laboratories (University of Minnesota, Iowa State University, and South Dakota State University) between July-2014 and June-2020. The PEDV spike protein coding gene (S) sequences generated during the postepidemic period (July-2014 and June-2020) across the U.S. (*n* = 96) with sampling location metadata (i.e., state, available for ~ 80% of the sequences) were obtained from the MSHMP database. The sequences were aligned with a set of S-INDEL variant references (GenBank accession number: KJ645635–708) [5] using the pairwise local alignment algorithm in MAFFT v.7.310 [17]. S-INDEL variants in the alignment were identified and differentiated from the U.S. prototype (nonS-INDEL) variants by comparing positions of insertion and deletion with the references and the INDEL profile documented elsewhere [18].

### 2.2. Statistical Analysis

#### 2.2.1. Spatiotemporal Analyses

To describe the spatial and temporal occurrence of PED cases on breeding farms, we performed a SaTScan analysis using SaTScan version 9.7 for each region. PEDV occurrence data was split into cases (when a farm reported the occurrence of a PEDV case) and controls (when a farm was reported as PED negative), encoded as 1 and 0, respectively. Subsequently we used the cases to perform spatial–temporal and seasonal permutations to identify clusters in PEDV outbreaks in time and space. For space-time clusters, we used 999 replications of the Standard Monte Carlo model for space-time permutation, scanning for high- and low-rate clusters with a 1-month time aggerate for the cases. The spatial window was set to constitute no more than 25% of the population at risk while temporal windows were set to the minimum and maximum sizes of at least 1 and no more than 6 months' worth of the study period [19], and the criteria for reporting secondary clusters was set to avoid geographical overlap. High-rate clusters were set up to include a minimum of five cases. No spatial or temporal adjustments were made on the data. Clusters were defined in a circular window for visualization. For seasonal cluster analysis, we used a discrete Poisson model with a monthly time aggregate of the cases [20]. Similar to the space-time permutation model, the minimum and maximum cluster sizes would constitute at least 1 month and no more than 25% worth of the study period and high-rate clusters had to have at least five cases. For all three analyses, the significance level cutoff for identified clusters was *p* ≤ 0.05.

#### 2.2.2. Risk Factor Analysis

To assess factors associated with observed occurrence of PEDV cases on the farms, we conducted a survival analysis with model covariates as farm/herd, region, use of air filtration systems, epidemiological year, farm size, county farm density, and season, with hazard (risk) of the PEDV case occurring as the outcome. In this model setup, dates were converted to a numeric format representing the number of days since the start of the study period to facilitate survival analysis. Categorical variables such as air filtration type, region, farm density, farm size, use of feed mitigants, and season were converted to factors. There were no missing values in the data. Using the Andersen–Gill model [21], a variation of the commonly used cox proportional hazards model, that allows for recurrent events in survival analysis, we modeled the time until the occurrence of PEDV cases and assessed the relationship between the survival time and various covariates. In the Andersen–Gill model, multiple records were created for each subject (farm/herd) for which an event occurred (PEDV case reported). Each of those records were then considered as separate episodes of a PEDV case with distinct temporal windows and hazard ratios estimated within the episodes [21]. The survival object was created using the *‘Surv'* function in R, with time measured in days since the start and events representing PEDV cases. The final model's goodness of fit and significance of covariates were assessed through summary statistics, Wald tests, and likelihood ratio tests. Data management was done in Microsoft Excel and preparation, and all analysis and modeling were done using R v4.2.1 [22] and relevant packages, including *survival* [23], and others, were utilized for modeling and visualization.

## 3. Results

A total of 621 PEDV cases were recorded in 372 farms in the study period (June 2014–July 2021). For the spatial–temporal analysis, all cases were included in checked analyses. In the risk factor analysis, where farms with no data on feed mitigant use were excluded, the number of cases reduced to 483 reported in 265 farms during the study period.

**Figure 3:**
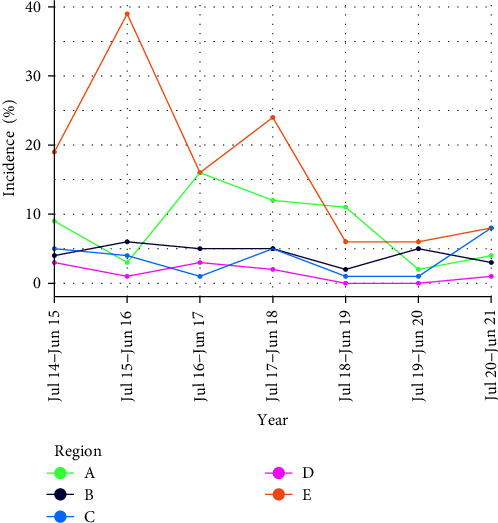
Regional cumulative incidence of PEDV occurrence per epidemiological year in U.S. breeding herds participating in the Morrison Swine Monitoring Project (MSHMP) between July 2014 and June 2021 (*N* = 86,267 observations [farm-weeks] from 1089 herds).

Overall, there was a spatial contraction of PEDV occurrence over the study period; cases were reported in 95 farms in 32 counties (11 states) in the first epidemiological year (July 2014–June 2015) and in 53 farms in 28 counties (9 states) in the last epidemiological year (July 2020 and June 2021). There was also a decline in the yearly cumulative incidence (# positive farms/population at risk) of PEDV in the study population across time, with the mean cumulative incidence declining from 8% ± 6.5% in the first epidemiological year to 4.8% ± 3.1% in the 7^th^ and final epidemiological year. The overall cumulative incidence for the entire study period was 6.9% ± 7.9%. This decline in incidence was more obvious in regions A and E than it was in the other regions (Figure 3).

**Figure 4:**
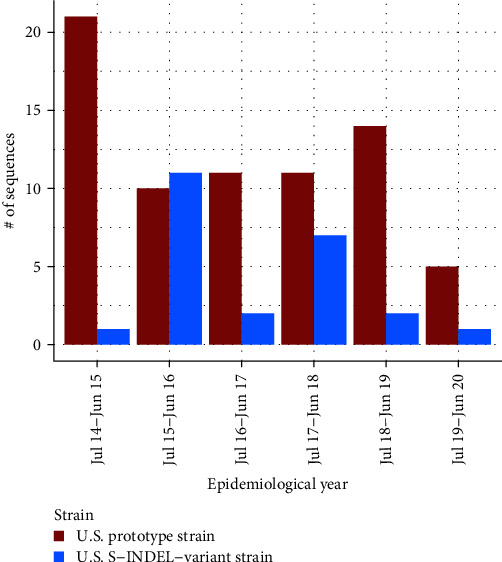
Frequency of distribution of PEDV strains reported in breeding herd participating in the Morrison Swine Health Monitoring Project (MSHMP) in the U.S. between July 2014 and June 2020.

From the five regions with outbreaks (A–E), 96 sequences were submitted to MSHMP between July-2014 and June-2020, 72 were the typical U.S. prototype strain while 24 were classified as the S-INDEL variant. Both strains were present in 6/7 epidemiological years considered in this study. Except for the second epidemiological year, the majority of the sequences were non-INDEL strains (Figure 4). Geographically, both strains were present in three of the five regions with the nonS-INDEL being the most abundant in all regions.

### 3.1. Spatiotemporal and Seasonality Analysis

From the seasonal cluster analysis, we identified that outbreaks were clustered seasonally (primarily in the winter, between December/January and March) throughout the study period with a relative risk of 2.2 (*p*=0.001). Additionally, there were 10 significant (*p* ≤ 0.03) spatiotemporal high-rate clusters identified from the spatial–temporal permutation analysis during the study period. The majority of the clusters (80%) occurred between 2015 and 2019, involving farms from 16 swine production systems. The clusters size ranged from as localized as 2.5 km^2^ to as large as 833.7 km^2^ (Table 1). The median size was 72.8 km^2^ with an interquartile range of 33.3–191.1 km^2^.

### 3.2. Risk Factor Analysis

A third of the farms in this study (190/562) reported using air filtration systems in their barns for part or the entire period of the study. Use of feed mitigants was not a widespread practice among farms whose data was available; only 5.3% (30/562) of herds reported using feed mitigants at some point during the study period. The mean ± SD farm density in the study area was 32 ± 30 farms/1000 km^2^ ranging from 2 to 2100 farms/10,000 km^2^. Regional mean ± SD farm densities were 4 ± 4, 30 ± 30, 20 ± 10, 30 ± 20, 50 ± 40 farms/1000 km^2^ for regions A–E, respectively. In this dataset, farm distribution by region was *A* = 114, *B* = 446, *C* = 112, *D* = 92, and *E* = 325. Breeding herd inventory during the study period ranged between 25 and 16,500 sows with a mean of 3,024 ± 1,901 (SD) and a median of 2600 sows. Due to noncompliance with the linearity assumption for risk factor modelling, these continuous parameters were categorized based on quantile splits and were fitted as three level factors, i.e., small farm (≤2000 sows), medium sized farm (2000–3500 sows), large farm (3500 sows) for farm inventory, and <13 farms, 13–31 farms, >31 farms per 1000 km^2^ for low, medium, and high farm density, as mentioned in the methods.

**Figure 5:**
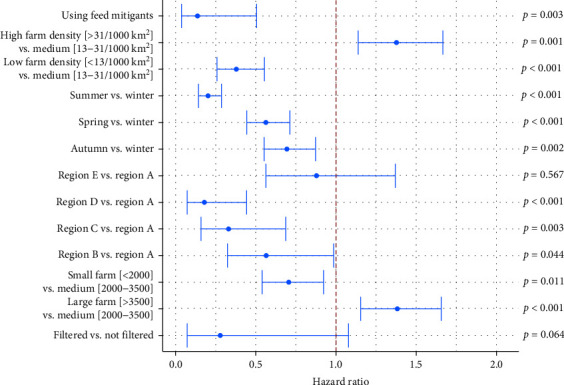
Summary of survival model analysis highlighting risk associated with factors influencing occurrence of PEDV cases on U.S. swine farms between July 2014 and June 2021.

In the model, all model covariates used in this analysis except epidemiological year had statistically significant associations with survival time (days until a case is reported). This model included 44,960 farm-weeks and 483 events/cases from 562 herds. The model had good discriminatory power to distinguish between farms where cases were reported earlier or later; the model had a concordance of 0.74 (se = 0.01) and significant Wald and likelihood ratio *p* values (<0.001), indicating improved fit of the model containing covariates compared to a null model. In this model, the risk of PEDV occurrence was smaller for farms in areas with lower county farm density. Compared to farms located in medium-density areas (13–31 farms/1000 km^2^), farms in low density areas had significantly lower risk for PEDV occurrence (hazard ratio = 0.4) while those in high density areas had higher risk of PEDV occurrence (hazard ratio = 1.4, *p*  < 0.001; Figure 5 and Table S1). Use of air filtration systems on farms also had a marginally protective effect, with filtered farms being at a lower risk of reporting a PEDV case with a hazard ratio of 0.3 (*p*=0.06). Addition of feed mitigants in pig diets had a protective effect, with farms using feed mitigants being at a significantly lower risk for PEDV occurrence (hazard ratio = 0.1, *p*=0.003) compared to farms where mitigants were not used (Figure 5 and Table S1). Winter was associated with the highest risk for PEDV cases amongst all seasons. Finally, all farms except for those in region E had significantly lower risk of PEDV occurrence than those in region B (Figure 5 and Table S1. This risk profile associated with season and region was in line with timing of spatiotemporal and seasonal clusters identified in the SaTScan analysis. Furthermore, consistent with the descriptive summary and the spatiotemporal analysis, there was an overall decrease in the risk of PEDV across years, although the variable was not significant and had exceptionally large confidence intervals, and hence was excluded from the final model reported here.

## 4. Discussion

After biosecurity and disease mitigation measures had an apparent effect on containing the PED epidemic circa summer 2013 into early 2014 in the U.S., a significant decline in the PEDV occurrence in summer 2014 was recognized as the end of the epidemic. Since then, we show that the overall incidence risk and spatial extent of PEDV cases has declined, with fewer breeding farms reporting cases and fewer states and counties experiencing outbreaks on their farms. However, PEDV continues to occur in U.S. swine herds with a cumulative annual incidence below 10% and with occasional seasonal increases in outbreaks [10]. In this study the cumulative incidence was around 7% with regional and temporal variation. Primarily, PEDV cases were 1.4 times more likely to occur on farms located in high swine density areas and large-sized farms (>3500 sows) compared to medium density areas and medium-sized farms. Additionally, there was statistical evidence that the use of feed mitigants was significantly associated with a reduced risk of PEDV occurrence (hazard ratio = 0.14; Figure 5 and Table S1).

Moreover, we observed that PEDV outbreaks clustered seasonally with twice as high odds for cases to occur in winter months. Spatiotemporally, clustering of PEDV cases sometimes involved more than one system in a given region, underscoring the multisystem challenges in PEDV management.

Like PRRS [24], the influence of seasonality in the occurrence of PEDV on farms has been suggested in the postepidemic period. Our study indicates that indeed, most breeding herd outbreaks of PEDV occur during the winter months. This increased occurrence of PEDV on farms could be as a result of increased virus better viability at cooler temperatures [25–28], lack of biosecurity compliance, mortality and manure management. Given that animal transportation equipment and personnel have been identified as potential areas of biosecurity breaches [29], research on seasonal variation in risk associated with animal transportation equipment and personnel could be explored in future studies.

Overall, there was concurrence between the spacetime permutation analysis and the risk factor analysis. Significant high-rate clusters were present in four of the six regions, with some regions recording more clusters and more farms from multiple systems than other regions. From the SaTScan analysis, region E had the highest number of cases, greatest number of clusters especially in earlier years, which was also consistent with the overt decline in incidence over the epidemiological years in that region (Figure 3). Region E was the only region for which the 95% confidence interval for the risk of PEDV occurrence (hazard ratio) was greater than 1, although not statistically significant from region A while all other regions had significantly lower risk for PEDV occurrence compared to region B. High farm density can increase the likelihood of windborne or so-called local area spread [30–32]. The overall observed decline in incidence risk in this region and nationally could be testament to PEDV-specific and more general efforts made by production systems to improve farm biosecurity and decrease the chances of viral introduction into breeding farms, such as air filtration systems [33, 34], feed ingredient quarantines [35], truck hygiene [30], among others.

The overall decline in spatial and temporal occurrence of PEDV (Figure 3 and Table 1) is encouraging and could point to an organic/systematic move towards possible elimination of PEDV cases in U.S. swine herds. As such, efforts to continually ensure that fewer cases occur or even conversations to establish counties or states free from PEDV may be on the horizon along with increased industrywide awareness and possible traction towards eliminating PEDV in the U.S. The dividends of these efforts are visible from the reduced economic losses from PEDV in US swine herds, from more than $1 billion during the epidemic [36, 37] to ~$50 million annually [38] which could be avoided with disease elimination. However, there is need to reevaluate these costs through robust studies.

During the early stages of the PEDV epidemic in the U.S., feed had been reported to potentially play a role in the transmission of PEDV [1, 6]. Therefore, the use of feed mitigants has been proposed as a potential PEDV risk mitigation measure [39, 40]. Throughout the study period, different feed mitigants regimes were implemented on these farms. Some systems reported never using feed mitigants on any of their farms, and some farms reported using mitigants for only part of the study period. Other systems used feed mitigants only on some farms perceived to be located in high-risk areas or farms housing high genetic level herds. In our study, although the use of feed mitigants was not differentiated by type of mitigant or method of application, the variation in when mitigants were used was captured in the data. Consistent with earlier, (experimental) studies [40], these data suggest that indeed using feed mitigants reduced the risk of PEDV occurrence on farms. Studies on feed mitigants primarily report a better clinical picture and performance in herds where feed mitigants were used compared to where they were not [40] and more resilient performance of the herds postexposure to disease. A decrease in the risk of outbreaks has not previously been overtly demonstrated. However, further research to quantify the preventive effects of mitigants to PEDV and better distinguish between hazard profiles associated with feed mitigants is needed to elucidate the protective effects of feed mitigants on PEDV occurrence observed in this study. For example, it is possible that the use of feed mitigants is confounded with other biosecurity practices that were not measured in this study. Experimental studies may be better suited to adequately address this question.

Use of air filtration systems significantly reduced the risk for PEDV occurrence on farms. While filtration has been documented to contribute to reduce the risk of PRRSV introduction, it is a resource-intensive biosecurity measure especially for a disease like PEDV whose transmission is mainly fecal-oral [3]. It is often assumed that farms investing in air filtration already have other parallel biosecurity practices in place to reduce disease introduction. In this case, we can infer that having an air filtration system functioned as a proxy to overall biosecurity practices of these farms, and thus farms with enhanced biosecurity practices would generally be less likely to break with PEDV. In connection with that, biosecurity management on large farms is likely to be more challenging than smaller or medium-sized farms. As such, the higher risk observed from the risk factor analysis likely speaks to other attributes associated with farm size that warrant further investigation with regards to PEDV epidemiology and management. Most of the observed associations between the different factors and the risk of PEDV on swine farms could be true for other swine diseases such as PRRS and *Mycoplasma hyopneumoniae* (and other bacterial and viral infections) which could co-occur in swine herds. Overall, although the specifics of disease spread could vary, certain biosecurity practices that disrupt pathogen transmission like air filtration, manure management, use of feed additives among others could be beneficial in management of PEDV, and other endemic diseases. Research specific to those disease conditions would better inform management and mitigation decisions and interventions.

As is expected with most observational studies, the interpretation of these findings should be done with consideration of some caveats. Firstly, during the data cleaning process, some observations were excluded from the data either because data on feed mitigants was not available for some systems and or some other metadata such as location was missing. Although there was a risk for introducing some bias in the data at this cleaning stage, these processes were necessary for accurate assessment of PEDV occurrence and associated risk factors. We mitigated any possible effects of data exclusion by ensuring that we used all viable data for spatiotemporal analysis and only excluded observations primarily based on missing feed mitigant data for the risk factor analysis. Additionally, the analytical approach for risk factor analysis ensured as much of the data was used and the overall alignment between the results from the two analyses (cluster and risk factor analysis) suggests, for example, that the data management procedures did not result in obvious loss of statistical power or bias. Secondly, farm density data was obtained from USDA statistics databases from one timepoint in the study. While we did not expect huge changes in farm density within our study period, the accuracy of the obtained data would have been better if actual farm records were available for each county in each epidemiological year.

## 5. Conclusion

Overall, we observe that although PEDV cases in the U.S. breeding herd are clustered in the winter months, the overall number of spatial clusters and their frequency has been declining across the country over time in this postepidemic phase. Additionally, there has been a general decline in incidence risk of PEDV outbreaks on farms in all regions over time, reflecting the impact of biosecurity and disease mitigation measures employed on the farms. As such, it may be timely to enhance those interventions and involve industry stakeholders in conversations of gradual elimination of PEDV in U.S. breeding herds and also the application of similar efforts in growing pig farms. Additionally, from our analysis, use of feed mitigants and use of air filtration systems were associated with reducing the risk of PEDV at farm level. Sustaining current effective PEDV mitigation strategies along with improved herd health may be a worthwhile course of action for long-term success in eliminating PEDV. Initial steps towards elimination may require an integrated regional approach with these practices along with other biosecurity measures. These study findings could also be more informative when augmented with more current data although reports indicate that PEDV incidence has not changed in the last 3 years (since 2021).

## Figures and Tables

**Table 1:** Summary of spatial–temporal permutation analysis scanning for high rate clusters of PEDV outbreaks among breeding farms participating in the Morison Swine Health Monitoring Project (MSHMP) in the U.S. between July 2014 and June 2021.

Region and cluster number	Time (month–year)	Area (km^2^)	Observed vs. expected	# of systems represented in cluster	Systems at risk	# of farms in cluster
A.1	Dec-17 to Feb-18	250.8	5.2	3	14	14
B.1	Jan-16 to Apr-16	833.7	8	6	27	34
B.2	Dec-15 to Feb-16	205	7.2	7	27	33
C.1	Feb-21 to Mar-21	149.4	3.1	1	11	10
E.1	Feb-17 to Mar-17	56.8	19.3	1	7	15
E.2	Dec-16 to Jan-17	2.5	33.8	1	7	5
E.3	Jan-15 to Mar-15	30.5	11.7	2	7	22
E.4	Nov-15 to Dec-15	88.7	11.7	4	7	54
E.5	Nov-19 to May-20	13.2	10.5	2	7	13
E.6	Oct-16 to Feb-17	42	14.5	1	7	6

## Data Availability

The data that support the findings of this study are not publicly available and are protected by confidentiality agreements.

## Supporting Information

Supporting Information Table S1: summary of cox proportional hazard model highlighting risk associated with factors influencing occurrence of PEDV cases on U.S. swine farms between July 2014 and June 2021.
