# Supplementary material for: Postepidemic Epidemiology of Porcine Epidemic Diarrhea Virus in the United States
Source: Transbound Emerg Dis. 2024 Oct 1;2024:5531899. doi: 10.1155/2024/5531899 (PMC12017030; doi:10.1155/2024/5531899)
Supplement: Supporting Information — Table S1: summary of cox proportional hazard model highlighting risk associated with factors influencing occurrence of PEDV cases on U.S. swine farms between July 2014 and June 2021. [file 5531899.f1.docx]

**Supplementary material**

| **Variable** | **Level** | **Exp(coef)** | **Exp(-coef)** | **95% Conf. Interval** | **P-value** |
| --- | --- | --- | --- | --- | --- |
| Air filtration | Not filtered | ref |  |  |  |
|  | Filtered | 0.28 | 3.59 | 0.07, 1.08 | 0.064 |
| Average inventory | Medium | ref |  |  |  |
|  | Small | 1.38 | 0.72 | 1.15, 1.66 | <0.001 |
|  | Large | 0.71 | 1.42 | 0.54, 0.92 | 0.011 |
| Season | Winter | ref |  |  |  |
|  | Fall | 0.69 | 1.44 | 0.55, 0.87 | 0.002 |
|  | Spring | 0.56 | 1.78 | 0.44, 0.71 | <0.001 |
|  | Summer | 0.20 | 4.94 | 0.14, 0.29 | <0.001 |
| Region | A | ref |  |  |  |
|  | B | 0.57 | 1.77 | 0.32, 0.99 | 0.044 |
|  | C | 0.33 | 3.03 | 0.16, 0.69 | 0.003 |
|  | D | 0.18 | 5.57 | 0.07, 0.44 | <0.001 |
|  | E | 0.88 | 1.14 | 0.56, 1.37 | 0.567 |
| Use of feed mitigants | No | ref |  |  |  |
|  | Yes | 0.14 | 7.31 | 0.04, 0.50 | 0.003 |
| County farm density | Medium | ref |  |  |  |
|  | Low | 0.38 | 2.64 | 0.26, 0.55 | 0.000 |
|  | High | 1.38 | 0.73 | 1.14, 1.67 | 0.001 |

Supplementary table 1: Summary of Cox proportional hazard model highlighting risk associated with factors influencing occurrence of PEDV cases on U.S. swine farms between July 2014 and June 2021.
